# Cross‐sectional study on knowledge and attitude of telemedicine in medical students of Nepal

**DOI:** 10.1002/hsr2.532

**Published:** 2022-02-18

**Authors:** Bijay Kunwar, Ayushma Dhungana, Binay Aryal, Arjun Gaire, Aramva Bikram Adhikari, Rajeev Ojha

**Affiliations:** ^1^ Department of Medicine Maharajgunj Medical Campus, Tribhuvan University Teaching Hospital Maharajgunj Nepal; ^2^ Department of Medicine National Medical College, National Medical College and Teaching Hospital Birgunj Nepal; ^3^ Department of Neurology Tribhuvan University Institute of Medicine Maharajgunj Nepal

**Keywords:** medical curriculum, medical education, medical students, Nepal, telehealth, telemedicine

## Abstract

**Background and Aims:**

Telemedicine is the delivery of healthcare services from distance using information and communication technology. It helps in overcoming the geographical physical barrier, increasing access to the healthcare services. Telemedicine has been growing in Nepal, with several hospitals and medical organizations providing services since 2006. COVID‐19 pandemic ignited significant interests in it, which previously remained unnoticed, realizing its importance for the present and future. The objective of this study was to evaluate the knowledge and attitude toward telemedicine among medical students in Nepal.

**Methods:**

An observational study among medical students in 19 medical colleges in Nepal was performed from May to June using Google forms. The survey consisted of 27 questions including information on demography and telemedicine exposure, its status in Nepal, interest, and plans for its future utilization.

**Results:**

Of 146 total surveyed students, 77.4% (n = 113) provided their views regarding telemedicine. Among students with knowledge of telemedicine, only 8.8% (n = 10) had attended some training. Only 6% (n = 4) of those who had previously consulted through telemedicine labeled their experience as poor. Exactly 88.5% (n = 100) were not satisfied with present telemedicine practices and coverage and 43.4% (n = 49) were optimistic about using telemedicine in future. Irrespective of location of medical colleges (Kathmandu Valley or outside) or levels of study (preclinical or clinical), students had similar knowledge and attitude regarding telemedicine.

**Conclusion:**

The participants have good knowledge regarding the importance of telemedicine but only few of them are educated regarding its usage. Despite limited exposure to telemedicine, participants advocate for expansion and wide use of telemedicine due to economy, technological advancement, and topographic diversities. Internet, sense of reliability, privacy issue, and lack of proper curriculum seem to have raised question on their positive attitude. Formal and structured education may enable optimistic aspirants to integrate telemedicine skills with medical care delivery with ease.

## 1 INTRODUCTION

Telemedicine is the use of electronic information and communication technologies to provide and support health care when distance separates the participants.^1^ It focuses on improving health outcomes by overcoming geographical barriers. Remote regions around the world face barriers in providing healthcare services, but recent advances in technology have made telemedicine possible.^2^ Its popularity in resource‐poor countries has been rising lately.^3^, ^4^, ^5^ The use of telemedicine has been constantly growing, with 44% increase in the years 2015 to 2019.^6^ During the first quarter of 2020, its use peaked by 50% in the United States compared with the same period in 2019.^7^

Nepal is a country with high Himalayas, green hills, and plain land. As beautiful as the country is with diversities, the barriers created by these diversities also prevail. This has not spared the healthcare system either and the topography has made it difficult for the health services to be accessible to every part of the country.^8^ Most of the healthcare workers prefer to work in urban areas, causing a shortage of healthcare services in the rural areas.^9^ Nepal also has inadequate number of trained health professionals, leading to a low doctor‐to‐patient ratio (0.7486 physicians per 1000 people in 2018).^10^ Furthermore, in the COVID‐19 pandemic, when the country is under restrictive measures such as physical distancing and even rigid lockdown, telemedicine can be a promising solution for most of these barriers.^11^, ^12^, ^13^ The past year has shown how beneficial telemedicine can be not just during the times of emergency, but also in the new normal.^14^ Although there are still challenges like digital literacy, infrastructure, and financing to be faced to implement telemedicine in Nepal,^15^ it has shown considerable promise to help the rural population both in Nepal and abroad.^15^, ^16^ In a country well known for its bureaucratic inefficiency, the prospect of lower costs and smooth operations are catered by telemedicine.^17^

The younger generation has undoubtedly more expertise in technology than the older generation and is regarded as the first generation of “digital natives.”^18^ As medicine continues to incorporate technology into the care of patients, it is important for medical institutions to expose the students to the modalities of care they will be utilizing in the future and to make them analyze and understand the merits and demerits of the same. For this, it is essential to first assess how much of telemedicine the future physicians know and have practiced. According to surveys performed among medical students globally, most of them either remain unfamiliar with telemedicine or have very limited understanding for the same throughout their course of study.^19^, ^20^, ^21^ The more clinical experience and education medical students have with telemedicine, the more positive their attitude on telemedicine becomes.^19^, ^21^, ^22^ In a cross‐sectional study performed in Austria, participants expressed moderate knowledge of eHealth and telemedicine concepts.^22^ In a descriptive, questionnaire‐based survey done in France, 50.7% of students and residents were in favor of telemedicine improving the patients' access to care, which may be due to the high familiarity thanks to the extensive national medical and non‐medical media discussions that took place before and during the release of the survey.^19^ Roughly two‐thirds of all online‐surveyed students in United States were undecided whether to utilize telemedicine in the future, which may be due to a deficit in the US medical schools' curricula in telemedicine leading to students' inability to articulate well‐informed opinions. This is supported by the fact that 86.5% of students were never exposed to telemedicine.^21^ There is no available literature yet that explores the presence of telemedicine in Nepal's medical curriculum, which makes it difficult to hypothesize the status of telemedicine knowledge and attitude in Nepali medical students. Similarly, no such assessment measuring the knowledge and attitude of telemedicine has been done among the students in Nepal.

Thus, this study is set out to evaluate the knowledge and attitude of telemedicine in medical students of Nepal.

## 2 METHODS

### 2.1 Study design and settings

An online questionnaire‐based survey was conducted from May 27, 2021, to June 21, 2021. The study population was a convenience (non‐probability) sample of medical students from all study levels (first to fifth years) of Bachelor of Medicine and Bachelor of Surgery (MBBS) from all 19 medical colleges of Nepal (n = 146), which helped to get a comprehensive understanding of the scenario throughout the country. The questionnaire was sent to participants via email. An informed consent was obtained via written format on the internet from the participants prior to proceeding with the questionnaire. The survey could only be filled once from a single email address, ensuring there was no double entry. Name and email address of participants were not recorded, ensuring anonymity. This descriptive cross‐sectional study was approved by Institutional Research Committee of Institute of Medicine (IOM) with reference number 428(6–11) E2077‐078.

The students undertaking MBBS affiliated to any University in Nepal were eligible to participate. The subjects unwilling to participate were excluded from the study. This self‐reporting nature of the study likely introduced voluntary response bias and Hawthorne bias. Hawthorne bias is the change in behavioral performance of research participants because they are aware of the observation, monitoring, assessment, or of the research itself.^23^

### 2.2 Questionnaire

Questionnaire was designed in an online survey platform, Google Forms. Questions were developed by adaptations of previously published literature.^19^, ^21^, ^22^ The survey consisted of 33 questions including demographic information (six items), information regarding telemedicine knowledge and exposure (12 items), opinions on telemedicine (12 items), and plans for future utilization (three items). Demographic information included age, gender, permanent address, level of study, medical college, and university of the respondents. Binary questions and multiple‐choice questions were used to assess the knowledge, exposure, and plans for future use of telemedicine. Under knowledge sub‐domain, the prior knowledge of the students regarding telemedicine was gauged with items 7 and 8 where students were asked to choose multiple answers, of which 3 were true and 2 were false. Items 9 to 20 measured the students' sources of telemedicine knowledge and experience of using telemedicine. Under attitude subdomain, the students' perspectives on the future use of telemedicine were taken. Respondents' attitude toward telemedicine were measured in items 23 to 33 on a five‐point Likert scale, ranging from 1 (strongly disagree) to 5 (strongly agree).

The survey was distributed via email to different student representatives at different medical colleges, who forwarded it to the students of their respective colleges via their own student email group channels to complete it at their own convenience, which may have introduced sample selection bias. A pretest was performed on 15 participating students prior to distributing the questionnaire to verify the comprehensibility of the data collecting instrument. Informed consent was taken from all the participants prior to the survey and confidentiality was maintained. The number of responses from a single user was limited to one.

### 2.3 Analysis

The data obtained from survey were entered in MS excel 2013 and exported to SPSS 26 (IBM Corp. Released 2018. IBM SPSS Statistics for Windows, Version 26.0 (Armonk, New York: IBM Corp.) for statistical analysis. Data were categorized according to the provincial address of students, the area of their colleges (inside or outside Kathmandu Valley), and the level of study (preclinical for first and second years/clinical for third, fourth, and fifth years).

Descriptive statistics included means, SDs, and frequency distributions. Fisher's exact test of independence was performed to evaluate differences in the data of different categories. A statistically significant difference was indicated by *P‐value* < .05.

## 3 RESULT

Of 146 people participating in the study, the average age of study participants was 21.89 ± 1.415 years. More than half of the participants were males, that is, 56.8% (n = 83). Participants from seven different provinces, which were formed on September 20, 2015, according to the Constitution of Nepal by grouping 77 districts, were included, consisting of 17, 29, 56, 16, 19, 1, and 8 from the provinces 1, 2, 3, 4, 5, 6, and 7 respectively. The highest number of participants were from third year (n = 94), followed by first (n = 20). Second and fourth years had equal number of participants (n = 14) and the least participation was from fifth year (n = 4). There were 112 students from clinical level, whereas 34 were from preclinical level. The study participants were from four different universities of Nepal. Kathmandu University (KU) had the highest number of participants (n = 64). Sixty‐two students participated from Tribhuvan University (TU), 11 from BP Koirala Institute of Health Sciences (BPKIHS), and nine from Patan Academy of Health Sciences (PAHS). Seventy‐two participants were from the colleges located inside Kathmandu Valley, whereas 74 were from colleges located outside the Kathmandu Valley.

**FIGURE 1:**
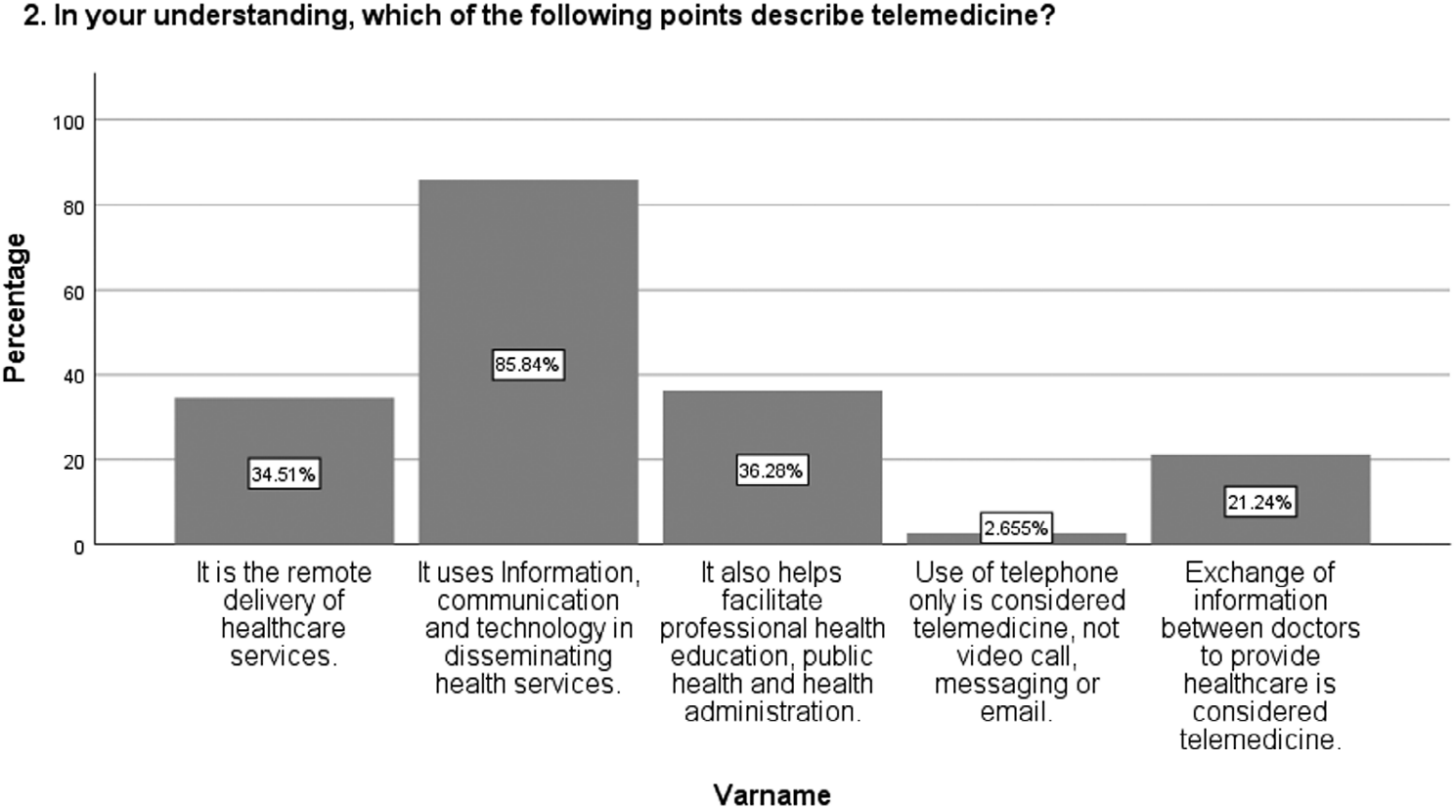
In your understanding, which of the following points describe telemedicine?

Among the participants, 22.6% (n = 33) answered that they had never heard the terms “eHealth,” “telemedicine,” or “Health Mobile.” From the 77.4% (n = 113) students who said that they had heard of at least one of the above terms, 85.84% answered “It uses information, communication and technology in disseminating health services,” 21.24% said “Exchange of information between doctors to provide healthcare is considered telemedicine,” and 34.51% described it as “The remote delivery of healthcare services,” all 3 of which were correct responses. See Figure 1 for more details.

Of 113 students, 26.5% (n = 30) said that telemedicine was practiced in their medical college hospital, 26.5% (n = 30) said it was not practiced, whereas 46.9% (n = 53) were unaware. When compared between universities, TU and KU showed similar percentages of students whose answers were “Yes” (25.5% vs 20.5%), “No” (29.4% vs 31.8%), or “I don't know” (45.1% vs 47.7%). (Table 1) Of 113 students, 2.3% (n = 3) said that telemedicine was included in their curriculum, which when analyzed according to universities, consisted of 1 (2%) in TU, 2 (4.5%) in KU, while 0 from BPKIHS and PAHS (Table 1). Among students with some knowledge of telemedicine (n = 113), only 8.8% (n = 10) had attended some training regarding telemedicine (Table 1).

**TABLE 1:** Students' response on knowledge and exposure of telemedicine

	Yes	No	Maybe/Do not Know	Total
	n (%)	n (%)	n (%)	N (%)
Do you know any telemedicine programs conducted in Nepal?				
Government	12 (10.6)	35 (31.0)	N/A	113 (100)
Private	37 (32.7)			
Both	29 (25.7)			
Does your medical college hospital practice telemedicine?				
	30 (26.5)	30 (26.5)	53 (46.9)	113 (100)
Is telemedicine included in the curriculum of your university?				
	3 (2.7)	63 (55.8)	47 (41.6)	113 (100)
Have you attended any training regarding telemedicine?				
	10 (8.8)	103 (91.2)	N/A	113 (100)
Have you ever consulted a doctor via telephone?				
	67 (59.3)	46 (40.7)	N/A	113 (100)
Will you recommend telephone consultation to your relatives and close friends?				
Regular	18 (26.9)	1 (1.5)	N/A	67 (100)
Emergency	48 (71.6)			
Do you know of any internet applications providing health services in Nepal?				
	42 (37.2)	71 (62.8)	N/A	113 (100)
If so, have you used the app?				
	13 (31.0)	29 (69.0)	N/A	42 (100)
Will you recommend this to your relatives and close friends?				
Regular	7 (53.8)	1 (7.7)	N/A	13 (100)
Emergency	5 (38.5)			
Do you plan to use telemedicine in the future in your clinical career?				
Occasionally	49 (43.4)	2 (1.8)	29 (25.7)	113 (100)
Frequently	33 (29.2)			
Are you satisfied with the amount of usage of telemedicine in Nepal currently?				
	13 (1.5)	100 (88.5)	N/A	113 (100)

Of 113 students, 59.3% (n = 67) had previously consulted a physician via telephone, whereas 40.7% (n = 46) had not (Table 1). The experience among those who had consulted before was measured, where 94% rated their experiences as satisfactory or better, with a mean Likert scale satisfaction score of 3.54 (Table 2). Also, of the 67 students with experience, 26.9% (n = 18) said they would recommend regular telephone consultation to their relatives and close friends (Table 1).

**TABLE 2:** Students' views on exposure to telemedicine

	Very poor	Poor	Satisfactory	Good	Very good	Total	Mean	SD
	n (%)	n (%)	n (%)	n (%)	n (%)	N (%)		
Experience of consultation over telephone	0 (0.0)	4 (6.0)	28 (41.8)	30 (44.8)	5 (3.4)	67 (100)	3.54	0.725
Experience of Using Telemedicine App	0 (0.0)	1 (7.7)	6 (46.2)	5 (38.5)	1 (7.7)	13 (100)	3.46	0.776

*Note*: Students' views on exposure to telemedicine.

Abbreviation: SD, standard deviation.

Of 113 students, 37.2% (n = 42) knew about internet applications providing health services in Nepal, out of which only 31.0% (n = 13) had used the app (Table 1).

The experience of those who had used the applications before was measured (Table 2). Also, 53.8% (n = 7) of the 13 said they would recommend regular use of those applications (Table 1).

Regarding the usage of telemedicine in their future clinical career, out of 113 students questioned, 43.4% (n = 49) planned to use it occasionally, 29.2% (n = 33) planned frequent usage, 1.8% (n = 2) did not intend to use it at all, whereas 25.7% (n = 29) were undecided. Also, regarding their satisfaction with the amount of usage of telemedicine in Nepal, currently 88.5% (n = 100) of them were unsatisfied (Table 1).

Of the 113 students who had heard about telemedicine, 41.1% (n = 60) agreed that telemedicine offers location‐independent services (3.49 ± 0.937). Also, 30.1% (n = 44) were neutral on reduction of multiple diagnoses, while 52.7% (n = 77) agreed that medical education in Nepal should include telemedicine in its curriculum (4.08 ± 0.696). See Table 3 for more details.

**TABLE 3:** Students' views on telemedicine

Strongly disagree	Disagree	Neutral	Agree	Strongly agree	Mean	SD
n (%)	n (%)	n (%)	n (%)	n (%)		
Telemedicine offers location‐independent health services						
6 (4.1)	9 (6.2)	30 (20.5)	60 (41.1)	8 (5.5)	3.49	0.937
Telemedicine reduces multiple diagnoses						
4 (2.7)	30 (20.5)	44 (30.1)	31 (21.2)	4 (2.7)	3.01	0.911
Online health information improves patient knowledge						
3 (2.1)	6 (4.1)	34 (23.3)	61 (41.8)	9 (6.2)	3.59	0.820
Quality of care is equal in telemedicine and face‐to‐face hospital visits						
30 (20.5)	63 (43.2)	13 (8.9)	6 (4.1)	1 (0.7)	1.98	0.824
Telemedicine improves interaction between physicians and patients and enhances doctor‐patient relationship						
1 (0.7)	24 (16.4)	31 (21.2)	54 (37.0)	3 (2.1)	3.30	0.865
Telemedicine reduces healthcare costs and administration						
1 (0.7)	3 (2.1)	18 (12.3)	76 (52.1)	15 (10.3)	3.89	0.686
Data security and privacy are guaranteed for electronically collected health data						
5 (3.4)	22 (15.1)	52 (35.6)	33 (22.6)	1 (0.7)	3.03	0.839
Collecting health data via telemonitoring improves the holistic view of the patients						
0	17 (11.6)	47 (32.2)	46 (31.5)	3 (2.1)	3.31	0.757
Telemedicine should replace nonessential real‐time appointments						
8 (5.5)	26 (17.8)	28 (19.2)	41 (28.1)	10 (6.8)	3.17	1.101
Telemedicine is essential in countries like Nepal						
2 (1.4)	4 (2.7)	21 (14.4)	71 (48.6)	15 (10.3)	3.82	0.770
The medical education system of Nepal should include telemedicine in its curriculum						
2 (1.4)	1 (0.7)	8 (5.5)	77 (52.7)	25 (17.1)	4.08	0.696

*Note*: Students' views on telemedicine.

Abbreviation: SD, standard deviation.

Of the preclinical students, 42.5% (n = 11) agreed (3.58 ± 0.89) that collecting health data via telemonitoring improves the holistic view of the patients, while 43.5% (n = 37) from clinical were neutral in response (3.23 ± 0.727) (*P* = .028). However, the responses to other views were quite similar regardless of the level of study in MBBS (Table 4).

**TABLE 4:** Students' views on telemedicine according to level of study

	Strongly disagree	Disagree	Neutral	Agree	Strongly agree	Mean	SD	*P* ^a^
	n (%)	n (%)	n (%)	n (%)	n (%)			
Telemedicine offers location‐independent health services								
Preclinical	2 (7.7)	4 (15.4)	6 (23.1)	13 (50.0)	1 (3.8)	3.27	1.041	.502
Clinical	4 (4.6)	5 (5.7)	24 (27.6)	47 (54.0)	7 (8.0)	3.55	0.899	
Telemedicine reduces multiple diagnoses								
Preclinical	1 (3.8)	7 (26.9)	10 (38.5)	5 (19.2)	3 (11.5)	3.08	1.055	.166
Clinical	3 (3.4)	23 (26.4)	34 (39.1)	26 (29.9)	1 (1.1)	2.99	0.869	
Online health information improves patient knowledge								
Preclinical	1 (3.8)	2 (7.7)	8 (30.8)	11 (42.3)	4 (15.4)	3.58	0.987	.309
Clinical	2 (2.3)	4 (4.6)	26 (29.9)	50 (57.5)	5 (5.7)	3.60	0.769	
Quality of care is equal in telemedicine and face‐to‐face hospital visits								
Preclinical	10 (38.5)	10 (38.5)	4 (15.4)	2 (7.7)	0 (0.0)	1.92	0.935	.230
Clinical	20 (23.0)	53 (60.9)	9 (10.3)	4 (4.6)	1 (1.1)	2.00	0.792	
Telemedicine improves interaction between physicians and patients and enhances doctor‐patient relationship								
Preclinical	1 (3.8)	4 (15.4)	5 (19.2)	15 (57.7)	1 (3.8)	3.42	0.945	.237
Clinical	0 (0.0)	20 (23.0)	26 (29.9)	39 (44.8)	2 (2.3)	3.26	0.842	
Telemedicine reduces healthcare costs and administration								
Preclinical	1 (3.8)	0 (0.0)	3 (11.5)	18 (69.2)	4 (15.4)	3.92	0.796	.462
Clinical	0 (0.0)	3 (3.4)	15 (17.2)	58 (66.7)	11 (12.6)	3.89	0.655	
Data security and privacy are guaranteed for electronically collected health data								
Preclinical	1 (3.8)	4 (15.4)	12 (46.2)	9 (34.6)	0 (0.0)	3.12	0.816	.924
Clinical	4 (4.6)	18 (20.7)	40 (46.0)	24 (27.6)	1 (1.1)	3.00	0.849	
Collecting health data via telemonitoring improves the holistic view of the patients								
Preclinical	0 (0.0)	2 (7.7)	10 (38.5)	11 (42.3)	3 (11.5)	3.58	0.809	.028 (*P < .05*)
Clinical	0 (0.0)	15 (17.2)	37 (42.5)	35 (40.2)	0 (0.0)	3.23	0.727	
Telemedicine should replace nonessential real‐time appointments								
Preclinical	2 (7.7)	7 (26.9)	9 (34.6)	5 (19.2)	3 (11.5)	3.00	1.131	.265
Clinical	6 (6.9)	19 (21.8)	19 (21.8)	36 (41.4)	7 (8.0)	3.22	1.094	
Telemedicine is essential in countries like Nepal								
Preclinical	1 (3.8)	1 (3.8)	5 (19.2)	15 (57.7)	4 (15.4)	3.77	0.908	0.777
Clinical	1 (1.1)	3 (3.4)	16 (18.4)	56 (64.4)	11 (12.6)	3.84	0.729	
The medical education system of Nepal should include telemedicine in its curriculum								
Preclinical	2 (7.7)	0 (0.0)	2 (7.7)	15 (57.7)	7 (26.9)	3.96	1.038	.113
Clinical	0 (0.0)	1 (1.1)	6 (6.9)	62 (71.3)	18 (20.7)	4.11	0.559	

*Note*: Students' views on telemedicine according to level of study.

Abbreviation: SD, standard deviation.

^a^ *P*: *P*‐value from Fischer's exact test.

Comparing first‐ and fourth‐year students, it was found that more percentage of fourth‐year students were provided with training regarding telemedicine (n = 3, 30%) than first‐year students (n = 1, 6.7%). It was also seen that the same percentage of first‐year students and fourth‐year students (80% each) planned to use telemedicine in the future, with just 1 (6.7%) first year and no fourth‐year students unwilling to do so. All students from final year (n = 4, 100%) had heard about telemedicine, while 25% of first‐year students (n = 5) had never heard about telemedicine. The students of fourth year agree more to telemedicine replacing non‐essential real‐time appointments than the first‐year students. The majority of students from each year feel a need to include telemedicine in the curriculum of Nepali medical education, consisting of 13 (86.7%) in first year, 9 (81.8%) in second year, 67 (91.8%) in third year, 9 (90%) in fourth, and 4 (100%) in fifth year.

Irrespective of location of medical colleges (Kathmandu Valley or outside valley), students had similar knowledge and attitude regarding telemedicine (Table 5).

**TABLE 5:** Students' views on telemedicine according to area of college

	Strongly disagree	Disagree	Neutral	Agree	Strongly agree	Mean	SD	*P* ^a^
	n (%)	n (%)	n (%)	n (%)	n (%)			
Telemedicine offers location‐independent health services								
Inktm^b^	3 (5.3)	4 (7.0)	12 (21.1)	32 (56.1)	6 (10.5)	3.60	0.961	.476
Outktm^c^	3 (5.4)	5 (8.9)	18 (32.1)	28 (50.0)	2 (3.6)	3.38	0.906	
Telemedicine reduces multiple diagnoses								
Inktm	2 (3.5)	13 (22.8)	23 (40.4)	15 (26.3)	4 (7.0)	3.11	0.958	.364
Outktm	2 (3.6)	17 (30.4)	21 (37.5)	16 (28.6)	0 (0.0)	2.91	0.859	
Online health information improves patient knowledge								
Inktm	2 (3.5)	4 (7.0)	18 (31.6)	29 (50.9)	4 (7.0)	3.51	0.869	.868
Outktm	1 (1.8)	2 (3.6)	16 (18.6)	32 (57.1)	5 (8.9)	3.68	0.765	
Quality of care is equal in telemedicine and face‐to‐face hospital visits								
Inktm	18 (31.6)	32 (56.1)	4 (7.0)	3 (5.3)	0 (0.0)	1.86	0.766	.389
Outktm	12 (21.4)	31 (55.4)	9 (16.1)	3 (5.4)	1 (1.8)	2.11	0.867	
Telemedicine improves interaction between physicians and patients and enhances doctor‐patient relationship								
Inktm	1 (1.8)	16 (28.1)	15 (26.3)	23 (40.4)	2 (3.5)	3.16	0.941	.229
Outktm	0 (0.0)	8 (14.3)	16 (28.6)	31 (55.4)	1 (1.8)	3.45	0.761	
Telemedicine reduces healthcare costs and administration								
Inktm	1 (1.8)	3 (5.3)	9 (15.8)	38 (66.7)	6 (10.5)	3.79	0.773	.366
Outktm	0 (0.0)	0 (0.0)	9 (16.1)	38 (67.9)	9 (16.1)	4.00	0.572	
Data security and privacy are guaranteed for electronically collected health data								
Inktm	4 (7.0)	13 (22.8)	22 (38.6)	17 (29.8)	1 (1.8)	2.96	0.944	.302
Outktm	1 (1.8)	9 (16.1)	30 (53.6)	16 (28.6)	0 (0.0)	3.09	0.721	
Collecting health data via telemonitoring improves the holistic view of the patients								
Inktm	0 (0.0)	10 (17.5)	25 (43.9)	20 (35.1)	2 (3.5)	3.25	0.786	.618
Outktm	0 (0.0)	7 (12.5)	22 (39.3)	26 (46.4)	1 (1.8)	3.38	0.728	
Telemedicine should replace nonessential real‐time appointments								
Inktm	5 (8.8)	17 (29.8)	10 (17.5)	18 (31.6)	7 (12.3)	3.09	1.214	.115
Outktm	3 (5.4)	9 (16.1)	18 (32.1)	23 (41.1)	3 (5.4)	3.25	0.977	
Telemedicine is essential in countries like Nepal								
Inktm	1 (1.8)	3 (5.3)	10 (17.5)	33 (57.9)	10 (17.5)	3.84	0.841	.555
Outktm	1 (1.8)	1 (1.8)	11 (19.6)	38 (67.9)	5 (8.9)	3.80	0.699	
The medical education system of Nepal should include telemedicine in its curriculum								
Inktm	2 (3.5)	0 (0.0)	5 (8.8)	36 (63.2)	14 (24.6)	4.05	0.811	.419
Outktm	0 (0.0)	1 (1.8)	3 (5.4)	41 (73.2)	11 (19.6)	4.11	0.562	

*Note*: Students' views on telemedicine according to area of college.

Abbreviation: SD, standard deviation.

^a^ *P*: *P*‐value from Fischer's exact test (Inktm vs Outktm).

^b^ Inktm: College Inside Kathmandu Valley.

^c^ Outktm: College Outside Kathmandu Valley.

## 4 DISCUSSION

This study, with the purpose of measuring the knowledge and attitude level of telemedicine in medical students, is the first of its kind in Nepal. The number of students who had heard of telemedicine (77.4%) was quite satisfactory, considering telemedicine is not yet universal and is still in a fledgling state in the country. The level of understanding of telemedicine among the students who had heard of it can be deemed as better than average. However, the percentage of students who did not consider telemedicine to be “remote delivery of healthcare services” or “exchange of information between doctors to provide healthcare”^13^ was relatively lower, which is understandable due to the low number of students having attended any sort of telemedicine training in their careers so far (8.8%). This figure was similar to the results of studies from the United States and France.^21^, ^24^ The COVID‐19 pandemic would have undoubtedly shifted the perspective toward telemedicine,^13^, ^16^, ^25^ and it would be interesting to see how these statistics evolve in the post‐pandemic world.

The level of awareness of the participants regarding government telemedicine programs in comparison to private programs was lower. The discontinuation of government telemedicine services due to apathy of the agencies under the health ministry may be a major reason.^26^ Majority of the participants said that they were not satisfied with the amount of usage of telemedicine in the country currently. While the government services have lagged behind, on the flipside, the COVID‐19 pandemic has resulted in promotion of various private telemedicine services, none more so than via picturesque infographics on various social media platforms, which has resulted in more than half the participants being aware of private telemedicine services. The number of students implying that telemedicine is included in their curriculum is almost nil, with the highest percentage being only 4.5% in KU. Although the curriculum of all undergraduate medical colleges under a single university is uniform, some individual colleges might have introduced telemedicine training programs, which led to a few students answering that telemedicine is included in the curriculum. This damning figure more or less confirms that the medical students in this country are devoid of receiving any sort of formal telemedicine education before they are thrust into their clinical practice. The comparison of responses between the groups of students in preclinical and clinical levels was performed to find out if there are any increments in the telemedicine experience of students at different levels of the medical curriculum. Also, the attitude of students could be gauged when they transition from classroom schooling to bedside teaching. The absence of telemedicine exposure to undergraduate medical students is further evidenced by the results in Table 4, showing no significant difference in the views regarding telemedicine between medical students of preclinical and clinical levels. This suggests that the attitude of students toward telemedicine does not change between preclinical and clinical level students. Introducing telemedicine courses to the curriculum can help in transforming opinions toward it when students reach clinical level of their study. This is certainly an issue that desires being addressed immediately, especially as the pandemic shows no signs of ending any time soon. Given the essential need for early clinical experiences to help medical students cultivate their skills, it follows that early implementation of telemedicine training at the undergraduate level will prepare future physicians for the telemedicine universe.^27^ There is unbalanced distribution of skilled health workers in Nepal^9^ and on top of it, seven medical colleges are clustered inside Kathmandu while all other colleges are the standalone medical college in their respective cities. Thus, the comparison between views of students studying in colleges inside Kathmandu and outside Kathmandu was done, but there were no significant differences between the two comparison groups. Again, the lack of telemedicine exposure and training could have led to the students being accustomed to the traditional healthcare delivery and unhinged by the potential of telemedicine regardless of the differing context of the location of their medical colleges.

A decent number of students had consulted a doctor over a telephone before (59.3%). A mean satisfaction rating of 3.54 among people who have consulted doctors over a telephone suggests that when applied, people have been quite satisfied with their telemedicine consultations and this bodes well for a promising future for telemedicine. It is supported by the fact that all but one of the participants were happy enough to recommend to their relatives and close friends to use the service. Although mobile telemedicine application usage was slightly lower, the satisfaction remained similarly encouraging, with the usage very likely to see a surge in the smartphone era.^28^ Regarded as the first generation of “digital natives,”^18^ this has translated to almost three‐fourths of medical students planning to use telemedicine in some capacity in their clinical careers. Even among the rest, there were far fewer students who flat out rejected to use telemedicine in the future rather than those who were undecided. With proper facilities of exposure and training, those on the fence can gain confidence and satisfaction to carry out healthcare efficiently via telemedicine.^29^, ^30^, ^31^ The association of job satisfaction with the use of telemedicine can also be a significant element to add to the list of benefits.^32^ Regardless, there is an indisputable argument that the medical education system of Nepal should include telemedicine in its curriculum, with a Likert scale mean of 4.08 obtained among the participants' answers.

The importance of telemedicine is further emphasized and there are compelling arguments that show why the participants agreed that telemedicine should be applied in a country like Nepal. The potential reduction in healthcare costs and administration is likely to be a huge factor in consideration and this is evidenced by the general agreement that telemedicine reduces healthcare costs (Table 3). It has the potential of being beneficial in low‐income countries like Nepal since it addresses the health problems of rural people who have difficulty in accessing healthcare services, and participants were seen to agree that telemedicine does provide location‐independent services.^2^ It is demonstrated that telemedicine use leads to timely access to health information, such as administrative and patients' records, diagnosis, and treatment profile records.^33^ On top of it, the possibility of avoiding hours of queuing for a medical consult, particularly in the current pandemic world, is a necessity rather than a luxury.^34^

Doctor‐patient relationship is under the microscope more than ever, especially in Asian countries, and miscommunications between doctors and patients is thought to be a common precursor.^35^ So, it was quite agreeable that doctor‐patients interactions could be improved with the use of telemedicine, as shown by the Likert scale mean of 3.30 obtained among the participant responses (Table 3). Also, with improved access to online information, patient knowledge on their disease can be improved so that medical counseling can become smoother. Confidentiality and security remain very important cogs in the wheel of health care regardless of the mode of service, and thus it is justifiable that there are worries when data are shared and stored in the internet.^28^, ^36^, ^37^ Although the concerns about data security and privacy were not particularly evident among medical students in this study, it will be fascinating to gauge the opinions of the patients and their caretakers on this matter as the technology becomes more and more ubiquitous.

Although these results unequivocally encourage the development of telemedicine in the country, the extent of its use remains a different conversation altogether. Among the participants with experience of consulting doctors over the phone, only 26.9% said that they would recommend it for regular appointments, suggesting that real‐time consultations are still preferred unless in cases of emergency, consistent with other available literature.^38^ Telemedicine should not be practiced as a substitute for conventional in‐person care but as a complementary service where traditional in‐person care is not feasible, accessible, or affordable. In numerous instances, clinical conditions of the patient may require higher detail of attention and may not be suitable via telemedicine. This is further elaborated by the general disagreement to the argument that quality of care is equal in telemedicine and face‐to‐face hospital visits (Mean = 1.84), and this opinion seemed constant regardless of the geographical location of medical colleges.

## 5 CONCLUSION

Through this study, efforts were made to measure the knowledge and attitude of telemedicine in medical students in Nepal for the first time. The results of this study suggest that although many students had heard of telemedicine before, most of them had not been exposed to formal telemedicine training and wanted to see it being included in the curricula. As a result, attitudes toward telemedicine hardly differed between students studying in different levels of medical college or different universities. With many upsides of telemedicine application in the context of a country like Nepal, on top of its usefulness during the COVID‐19 pandemic, it will be beneficial to introduce telemedicine training to students and apply it in clinical practice.

### 5.1 Limitations

This study only gauges the opinion of medical students regarding telemedicine, and it is important to evaluate the views of the healthcare recipients since they form the other half of doctor‐patient relationship. As the questionnaire of our study was disseminated via the internet, it may have shown sample selection bias toward the students with internet access. The number of students from each medical college and years of study was not uniform. The difference in number of students in comparison groups was due to non‐probability sampling causing unequal distribution. Due to it being an online questionnaire, the results may have shown voluntary response bias and Hawthorne bias. Hawthorne bias can be reduced by conducting offline surveys in natural settings restricting the use of sources of information.

## FUNDING

There are no sources of funding.

## CONFLICT OF INTEREST

There are no conflicts of interest.

## AUTHOR CONTRIBUTIONS

Conceptualization: Bijay Kunwar.

Formal Analysis: Bijay Kunwar.

Methodology: Bijay Kunwar.

Project Administration: Rajeev Ojha.

Resources: Ayushma Dhungana, Binay Aryal, Arjun Gaire, Aramva Bikram Adhikari.

Software: Ayushma Dhungana, Binay Aryal, Arjun Gaire, Aramva Bikram Adhikari.

Supervision: Rajeev Ojha.

Validation: Rajeev Ojha.

Visualization: Ayushma Dhungana, Binay Aryal, Arjun Gaire, Aramva Bikram Adhikari.

Writing—Original Draft: Bijay Kunwar, Ayushma Dhungana.

Writing—Review and Editing: Rajeev Ojha.

The corresponding author confirms that he had full access to all of the data in the study and takes complete responsibility for the integrity of the data and the accuracy of the data analysis.

## TRANSPARENCY STATEMENT

The manuscript is an honest, accurate, and transparent account of the study being reported; that no important aspects of the study have been omitted; and that any discrepancies from the study as planned (and, if relevant, registered) have been explained.

## ETHICS STATEMENT

The work being submitted has been done in accordance with Wiley's Best Practice Guidelines on Publishing Ethics, in an ethical and responsible way, with no research misconduct, which includes, but is not limited to data fabrication and falsification, plagiarism, image manipulation, unethical research, biased reporting, authorship abuse, redundant or duplicate publication, and undeclared conflicts of interest.

## References

[1] Telemedicine I of M (US) C on ECA of, Field MJ. Introduction and Background. 1996. https://www.ncbi.nlm.nih.gov/books/NBK45440/

[2] Jha AK , Sawka E , Tiwari B , et al. Telemedicine and community health projects in Asia. Dermatol Clin. 2021;39:23‐32. 33228859 10.1016/j.det.2020.08.003

[3] Bagayoko CO , Anne A , Fieschi M , Geissbuhler A . Can ICTs contribute to the efficiency and provide equitable access to the health care system in sub‐Saharan Africa? The Mali experience. Yearb Med Inform. 2011;6(1):33‐38. 21938322

[4] Humayun A , Shahabuddin S , Afzal S , Malik AA , Atique S , Iqbal U . Healthcare strategies and initiatives about COVID19 in Pakistan: telemedicine a way to look forward. Comput Methods Programs Biomed Update. 2021;1:100008. 34337588 10.1016/j.cmpbup.2021.100008 PMC8023788

[5] Dash S , Aarthy R , Mohan V . Telemedicine during COVID‐19 in India—a new policy and its challenges. J Public Health Policy. 2021;42:501‐509. 34012012 10.1057/s41271-021-00287-w PMC8131484

[6] Telehealth Services in the US ‐ Industry Data, Trends, Stats | IBISWorld.

[7] Koonin LM , Hoots B , Tsang CA , et al. Trends in the use of telehealth during the emergence of the COVID‐19 pandemic — United States, January–march 2020. MMWR Morb Mortal Wkly Rep. 2020;69(43):1595‐1599. 33119561 10.15585/mmwr.mm6943a3 PMC7641006

[8] Paudel M , Javanparast S , Newman L , Dasvarma G . Health system barriers influencing perinatal survival in mountain villages of Nepal: implications for future policies and practices. J Health Popul Nutr. 2018;37(1):1‐19. 29976245 10.1186/s41043-018-0148-y PMC6034263

[9] Morrison J , Shrestha NR , Hayes B , Zimmerman M . Mobile phone support for rural health workers in Nepal through “celemedicine”. JNMA J Nepal Med Assoc. 2013;52(191):538‐542. 24907968

[10] World Development Indicators | DataBank.

[11] Ting DSW , Carin L , Dzau V , Wong TY . Digital technology and COVID‐19. Nat Med. 2020;26(4):459‐461. 32284618 10.1038/s41591-020-0824-5 PMC7100489

[12] Greenhalgh T , Koh GCH , Car J . Covid‐19: a remote assessment in primary care. BMJ. 2020;368:1‐5. 10.1136/bmj.m1182 32213507

[13] Montelongo A , Becker JL , Roman R , et al. The management of COVID‐19 cases through telemedicine in Brazil. PLoS One. 2021;16(7):e0254339. doi:10.1371/journal.pone.0254339 34260644 PMC8279372

[14] Te Liao C , Chang WT , Yu WL , Toh HS . Utility of telemedicine in the COVID‐19 era. Rev Cardiovasc Med. 2020;21(4):577‐581. 33388003 10.31083/j.rcm.2020.04.188

[15] Bhatta R , Aryal K , Ellingsen G . Opportunities and challenges of a rural‐telemedicine program in Nepal. J Nepal Health Res Counc. 2015;13(30):149‐153. 26744201

[16] Wang Y , Yang J , Ma H , et al. Application of telemedicine in the COVID‐19 epidemic: an analysis of Gansu Province in China. PLoS One. 2021;16(8):e0249872. doi:10.1371/journal.pone.0249872 34347779 PMC8336882

[17] DeWyer A , Scheel A , Kamarembo J , et al. Establishment of a cardiac telehealth program to support cardiovascular diagnosis and care in a remote, resource‐poor setting in Uganda. PLoS One. 2021;16(8):e0255918. doi:10.1371/journal.pone.0255918 34358281 PMC8345851

[18] Pathipati AS , Azad TD , Jethwani K . Telemedical education: training digital natives in telemedicine. J Med Internet Res. 2016;18(7):e193. 27405323 10.2196/jmir.5534 PMC4961876

[19] Yaghobian S , Ohannessian R , Iampetro T , et al. Knowledge, attitudes and practices of telemedicine education and training of French medical students and residents. J Telemed Telecare. 2020;1:1357633X2092682. 10.1177/1357633X20926829 32517545

[20] Cornes S , Gelfand JM , Calton B . Foundational telemedicine workshop for first‐year medical students developed during a pandemic. MedEdPORTAL J Teach Learn Resour. 2021;17(1):11171. 10.15766/mep_2374-8265.11171 PMC8282677 34337148

[21] Kong SS , Azarfar A , Ashour A , Atkins C , Bhanusali N . Awareness and attitudes towards telemedicine among medical students in the United States. Cureus. 2020;12(11):1‐10. 10.7759/cureus.11574 PMC7749854 33364099

[22] Wernhart A , Gahbauer S , Haluza D . eHealth and telemedicine: practices and beliefs among healthcare professionals and medical students at a medical university. PLoS One. 2019;14(2):1‐13. 10.1371/journal.pone.0213067 PMC6394957 30818348

[23] Nguyen VNB , Miller C , Sunderland J , McGuiness W . Understanding the Hawthorne effect in wound research—a scoping review. Int Wound J. 2018;15(6):1010‐1024. doi:10.1111/iwj.12968 30136375 PMC7949616

[24] Yaghobian S , Ohannessian R , Mathieu‐Fritz A , Moulin T . National survey of telemedicine education and training in medical schools in France. J Telemed Telecare. 2019;26(5):303‐308. doi:10.1177/1357633X18820374 30602352

[25] Keesara S , Jonas A , Schulman K . Covid‐19 and health care's digital revolution. N Engl J Med. 2020;382(23):e82. doi:10.1056/NEJMp2005835 32240581

[26] Ministry of Health and Population to resume telemedicine services. https://kathmandupost.com/health/2019/09/20/government-to-resume-telemedicine-services

[27] Budakoğlu Iİ , Sayılır MÜ , Kıyak YS , Coşkun Ö , Kula S . Telemedicine curriculum in undergraduate medical education: a systematic search and review. Health and Technology. Berlin/Heidelberg, Germany: Springer Science and Business Media Deutschland GmbH; 2021:1‐9. 10.1007/s12553-021-00559-1 PMC8109844 33996380

[28] Kim E , Torous J , Horng S , et al. Mobile device ownership among emergency department patients. Int J Med Inform. 2019;126:114‐117. 31029252 10.1016/j.ijmedinf.2019.03.020

[29] Unrue EL , White G , Cheng N , Lindsey T . Effect of a standardized patient encounter on first year medical student confidence and satisfaction with telemedicine. J Osteopath Med. 2021;121:733‐737. doi:10.1515/jom-2020-0277 34192837

[30] Kirkland EB , DuBose‐Morris R , Duckett A . Telehealth for the internal medicine resident: a 3‐year longitudinal curriculum. J Telemed Telecare. 2019;27:599‐605. doi:10.1177/1357633X19896683 31888396

[31] Shayan W , Dicker AP . Telemedicine training in undergraduate medical education: mixed‐methods review. JMIR Med Educ. 2019;5(1):e12515. 30958269 10.2196/12515 PMC6475822

[32] Atinga RA , Abor PA , Suleman SJ , Anaba EA , Kipo B . E‐health usage and health workers' motivation and job satisfaction in Ghana. PLoS One. 2020;15(9):e0239454. 32966323 10.1371/journal.pone.0239454 PMC7510985

[33] Teviu EAA , Aikins M , Abdulai TI , et al. Improving medical records filing in a municipal hospital in Ghana. Ghana Med J. 2012;46(3):136‐141. 23661826 PMC3645163

[34] Hollander JE , Carr BG . Virtually perfect? Telemedicine for Covid‐19. 2020;382(18):1679‐1681. doi:10.1056/NEJMp2003539 32160451

[35] Kumari A , Kaur T , Ranjan P , Chopra S , Sarkar S , Baitha U . Workplace violence against doctors: characteristics, risk factors, and mitigation strategies. J Postgrad Med. 2020;66(3):149. 32675451 10.4103/jpgm.JPGM_96_20 PMC7542052

[36] Al‐Khalifa KS , Al SR . Teledentistry awareness among dental professionals in Saudi Arabia. PLoS One. 2020;15(10):e0240825. doi:10.1371/journal.pone.0240825 33057381 PMC7561132

[37] Shen N , Bernier T , Sequeira L , et al. Understanding the patient privacy perspective on health information exchange: a systematic review. Int J Med Inform. 2019;125:1‐12. 30914173 10.1016/j.ijmedinf.2019.01.014

[38] Chiu C‐Y , Sarwal A , Jawed M , Chemarthi VS , Shabarek N . Telemedicine experience of NYC internal medicine residents during COVID‐19 pandemic. PLoS One. 2021;16:e0246762. doi:10.1371/journal.pone.0246762 33556151 PMC7869991

